# Intracerebroventricularly Injected Streptozotocin Exerts Subtle Effects on the Cognitive Performance of Long-Evans Rats

**DOI:** 10.3389/fphar.2021.662173

**Published:** 2021-05-07

**Authors:** Attila Gáspár, Barbara Hutka, Aliz Judit Ernyey, Brigitta Tekla Tajti, Bence Tamás Varga, Zoltán Sándor Zádori, István Gyertyán

**Affiliations:** Department of Pharmacology and Pharmacotherapy, Semmelweis University, Budapest, Hungary

**Keywords:** Alzheimer disease model, STZ icv., cognitive test battery, learning impairment, β-amyloid, phospho-tau

## Abstract

Intracerebroventricularly injected streptozotocin (STZ)-induced learning impairment has been an increasingly used rat model of Alzheimer disease. The evoked pathological changes involve many symptoms of the human disease (cognitive decline, increase in β-amyloid and phospho-tau level, amyloid plaque-like deposits). However, the model has predominantly been used with Wistar rats in the literature. The objective of the current study was to transfer it to Long-Evans rats with the ulterior aim to integrate it in a complex cognitive test battery where we use this strain because of its superior cognitive capabilities. We performed two experiments (EXP1, EXP2) with three months old male animals. At EXP1, rats were treated with 2 × 1.5 mg/kg STZ (based on the literature) or citrate buffer vehicle injected bilaterally into the lateral ventricles on days 1 and 3. At EXP2 animals were treated with 3 × 1.5 mg/kg STZ or citrate buffer vehicle injected in the same way as in EXP1 at days 1, 3, and 5. Learning and memory capabilities of the rats were then tested in the following paradigms: five choice serial reaction time test (daily training, started from week 2 or 8 post surgery in Exp1 or Exp2, respectively, and lasting until the end of the experiment); novel object recognition (NOR) test (at week 8 or 14), passive avoidance (at week 11 or 6) and Morris water-maze (at week 14 or 6). 15 or 14 weeks after the STZ treatment animals were sacrificed and brain phospho-tau/tau protein ratio and β -amyloid level were determined by western blot technique. In EXP1 we could not find any significant difference between the treated and the control groups in any of the assays. In EXP2 we found significant impairment in the NOR test and elevated β-amyloid level in the STZ treated group in addition to slower learning of the five-choice paradigm and a trend for increased phospho-tau/tau ratio. Altogether our findings suggest that the Long-Evans strain may be less sensitive to the STZ treatment than the Wistar rats and higher doses may be needed to trigger pathological changes in these animals. The results also highlight the importance of strain diversity in modelling human diseases.

## Introduction

The bitter experience of anti-dementia drug development over the past 15 years has been that clinical trials of potential cognitive enhancers have resulted in 100% failure, mostly due to lack of efficacy (Cummings et al., 2014). One of the main reasons for the serial failures is the low translational value of animal experimental models predicting human efficacy. In the case of Alzheimer’s disease (AD) therapeutic approaches were based almost exclusively on the amyloid cascade hypothesis (Barage and Sonawane, 2015), and its key models were transgenic mouse lines carrying human mutant transgenes characteristic for the familial form of the disease. These strains are characterized by massive human β-amyloid overproduction, but this can be considered a model of amyloid intoxication rather than the disease itself, as they did not show tau pathology and the observed cognitive defects did not correlate with histological changes (Foley et al., 2015). The series of failures in clinical trials (Schneider et al., 2014) have raised serious doubts not only about the validity of the transgenic models but also about the validity of the amyloid theory itself (Herrup, 2015). For these reasons, non-transgenic models of sporadic AD have again become the focus of research. One prominent representative of these is the intracerebroventricularly (icv.) injected streptozotocin (STZ)-induced insulin-resistant brain state (Chen et al., 2013; Salkovic-Petrisic et al., 2013). The theoretical basis of the model is the cerebral insulin resistance in AD, which is why the disease is also referred to as type 3 diabetes (Chen and Zhong, 2013). As a result of insulin resistance induced by STZ treatment (Craft, 2006; Agrawal et al., 2011; De Felice et al., 2014), AD-like pathology develops (increased phospho-tau at 1 month post-injection, β -amyloid at 3 months, appearance of plaques at 6 months) associated with cognitive deficits (already at 1 month) (Knezovic et al., 2015). Based on the data to date, it appears to be a more adequate model than transgenic mice (Salkovic-Petrisic et al., 2013) and has the additional advantage of being applicable to rats.

Our group elaborated and established a rat cognitive test battery and testing protocol for more reliable prediction of clinical efficacy of putative cognitive enhancer drugs (Gyertyán, 2017; Gyertyán et al., 2020). According to the protocol, several cognitive tasks representing different cognitive domains were taught to the same cohort of Long-Evans rats, for example, five-choice serial reaction time task (5-CSRTT) for attention, a cooperation task for social cognition (Kozma et al., 2019), Morris water maze paradigm for spatial memory, “pot-jumping” exercise for procedural memory (Ernyey et al., 2019). Hereby we created a population with “widespread knowledge” (Gyertyán et al., 2016). The Long-Evans strain was chosen for its good learning capability, which is an essential requirement in a system imposing heavy cognitive load on the subjects. The effect of a particular impairment method on the various cognitive functions could then be simultaneously measured in this trained population. These impaired states served then as the target of potential cognitive enhancer treatments in a “clinical trial-like”, vehicle controlled, double blind, randomized experimental design (Gyertyán et al., 2020). The icv. STZ-model could be integrated into this testing protocol as a distinguished, particularly useful impairing method of high translational potential. As the model has been used with Wistar–and to a lesser extent Sprague-Dawley rats in the literature, transferring it into Long-Evans animals is the first step toward this integration. The objective of the current study was to try to reproduce the cognitive and biochemical changes described in Wistar rats in the literature in naive Long-Evans rats as well.

## Methods and Materials

### Animals

Eight-nine weeks old male Long-Evans rats (Janvier Labs, Le Genest-Saint-Isle, France) were used in this study; 18 subjects weighing 240–280 g in experiment 1 (EXP1), and 24 subjects (210–270 g) in experiment 2 (EXP2). Animals were kept three per cage (1376 cm^2^ polycarbonate cages with paper tubes and wooden bricks as environmental enrichment tools) under reverse light dark cycle (dark phase from 4 am to 4 pm). Food (commercial pellet rat feed R/M−Z + H produced by SSniff Spezialdiäten GmbH, Soest, Germany) was available *ad libitum* up to the end of the post-injection recovery period; after that the animals had a restricted food access: the amount of the food was 45 g for three rats and it was supplied before the end of the dark phase. Drinking water was available ad libitum over the whole course of the experiment. The animals were intensively handled before and during the experiments. At the end of the behavioral measurements, they were anaesthetized by isoflurane and decapitated to remove their hippocampus for the western blot measurements. The experiments were authorized by the regional animal health authority in Hungary (resolution number PE/EA/785–5/2019) and conformed to the Hungarian welfare law and the EU 63/2010 Directive.

### Intracerebroventricular Streptozotocin Treatment

**FIGURE 1:**
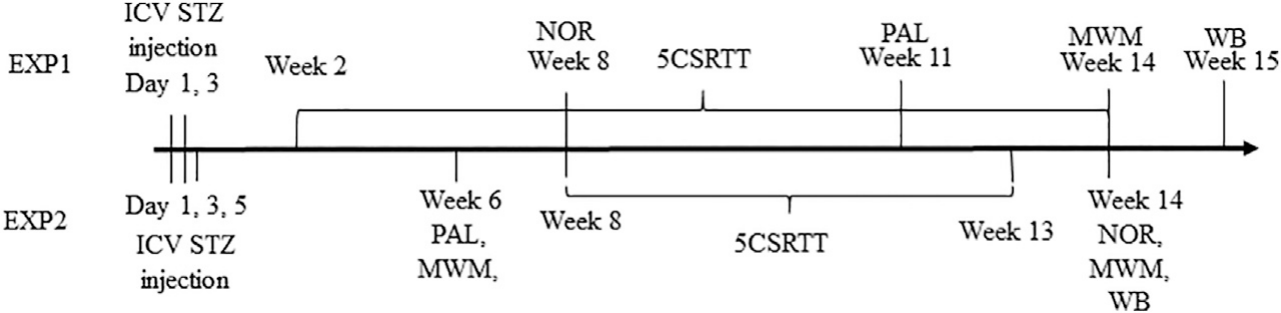
Timeline of the experiments (EXP, experiment; ICV, intracelebroventricular; STZ, streptozotocin; NOR, novel object recognition; 5CSRTT, five choice reaction time task; PAL, passive avoidance learning; MWM, Morris water maze; WB, western blot).

During EXP1, 3 mg/kg icv STZ (Sigma-Aldrich, St. Louis, MO, United States) divided into two 1.5 mg/kg doses were given bilaterally at day 1 and day 3. A volume of 2 μL/ventricle was injected to the left and the right ventricle for a rat of 500 g. The dose was adjusted to the body mass of the animal by changing the injection volume. At EXP2, rats were treated with 4.5 mg/kg STZ split into three equal doses administered on day 1, 3, and 5 (Figure 1). In both experiments, STZ was dissolved in 0.05 M citrate buffer pH 4.5 [sodium citrate dihydrate (0,0228 M) and citric acid (0,0272 M), Santa Cruz Biotechnology (Santa Cruz, CA, United States)]. The control groups received vehicle treatment in both experiments.

In EXP1, rats were anesthetized by sodium pentobarbital (60 mg/kg, i.p.) at both injections. Unfortunately, one animal from the control group could not recover from anesthesia. In EXP2, rats received anaesthesia via a mixture of ketamine (80 mg/kg) and xylazine (10 mg/kg ip.) during the first drug administration and isoflurane (4% in pure oxygen) during the 2^nd^ and 3^rd^ surgeries. Animals were placed in a stereotactic apparatus (Stoelting, Wood Dale, IL, United States) and laid on a heating bench (37°C) (Supertech Instruments, Pécs, Hungary). Midline incision on the skin was made and the surface of the skull was cleaned. Drilled holes at the place of the injection was made by dental drill. The ICV coordinates were: 0.72 mm posterior to Bregma, 1.5 mm lateral to sagittal suture, 3.6 mm ventral of the surface of the brain (Noble et al., 1967). A guide cannula was placed into the drilled hole in the skull and STZ was infused by a Hamilton syringe via a microinjection pump (CMA/100, CMA/Microdialysis Ab, Stockholm, Sweden); the injection speed was 5 min/hole. The needle was left in place for an additional 2 minutes then the guide canule was removed and the wound sutured. After the last treatment, the holes were closed by bone-cement. After the surgery, rats were given buprenorphine (0.05 mg/kg i.p.) and lidocaine was applied to the wound as analgesics. During the period of the surgeries and one week thereafter the animals had *ad libitum* food access. Until the wounds healed (approximately two weeks), the animals were kept separately.

### Behavioural Assays

#### Novel Object Recognition

The test apparatus was a 48x48x42 cm box with bedding material on the bottom where the behaviour of the animals were recorded by a video camera system. Before the testing day, rats were habituated to the test box for 3 minutes (EXP1) or 10 minutes (EXP2). The assay itself consisted of two trials, an acquisition trial and a retention trial. In the acquisition trial, the rats had 3 minutes to explore two identical objects in the box. The objects were placed 10 cm from the diagonally opposite corners and 40 cm from each other. After a delay of 80 minutes (EXP1) or 60 minutes (EXP2), in the retention trial one of the objects was changed to a novel one and the animals had 3 minutes again to explore them. The recognizable objects were a glass jar and a plastic jar in EXP1 and a plastic bottle filled with gravel and a glass bottle filled with blue dye solution in EXP2. Exploration time of each object was the registered parameter. Recognition memory was characterized by the discrimination index according to the following equation: $\mathrm{DI}=\frac{\mathrm{new object}-\mathrm{old object}}{\mathrm{new object}+\mathrm{old object}}\times \mathrm{100}$ . Animals which explored the objects for less than 10 seconds or explored only one of the two objects in any of the trials were excluded from the experiment (2 animals from the control group and one from the STZ group in EXP1, and one animal from the control group and two rats from the STZ group in EXP2).

#### Passive Avoidance Learning

The type of the experiment was a step through passive avoidance test. The apparatus consisted of a light and a dark chamber separated by a guillotine door. The test consisted of two parts, the acquisition trial and 24 hours later the retention trial. During the trials the rats were placed into the light chamber and 30 sec later the door opened and the animal could cross into the dark chamber. In the acquisition trial the animals had 180 sec (cut off time) to enter the dark compartment of the device, whereas at the retention trial the cut off time was 300 sec. When the rat passed through to the dark side, the door closed and after a 3 seconds delay a mild foot shock (0,6 mA, 3 sec) was delivered. The animal was left in the dark compartment for an additional 5 seconds after the shock. The measured parameters were entry latencies into the dark compartment in the acquisition and the retention trials.

#### Morris Water Maze

The apparatus was a black circular pool (diameter 190 cm, depth 60 cm) filled with water (38 cm, 23 ± 1°C) and containing a non-visible round escape platform (10 cm diameter) placed 0.5 cm below the water surface. The platform was located in the south-east (SE) quadrant, 40 cm from the edge of the pool. On the wall of the experimental room extra-maze cues were placed to facilitate the orientation during swimming. At the start of a trial the rat was placed into the pool at one of the four possible start points (North, East, West or South rotated in a systemic manner) had 3 minutes to find the hidden escape platform. When the animal didn’t find it, it was gently guided to the platform. Rats were allowed to spend 30 sec on the platform then were taken out, dried with a cloth and replaced in their home-cage. During the acquisition phase the animals were trained in 3 daily trials for two (EXP1) or three (EXP2) consecutive days. The interval between the trials was 30 min. Escape latency was measured and swimming path was recorded by Smart v3.0 video tracking system software (Panlab, Barcelona, Spain). Two days after the last acquisition trial, the animals were tested in a probe trial when the hidden platform was removed from the maze. In this measurement, the rats had 2 minutes to explore the maze, the measured parameter was the time they spent in the target quadrant (where the platform had been located during the acquisition trials). After a 30 min delay, the hidden platform was replaced to the maze at a different position [north-west (NW)], and 3 more acquisition trials were run. With the EXP2 group, 3 months after the STZ treatment an acquisition session (hidden platform located at NW) and two days after a single probe trial was made.

#### 5-Choice Serial Reaction Time Task

5CSRTT device consist of a 31x35x34 cm test box (cat. no. 259920) (TSE Systems, Bad Homburg vor der Höhe, Germany). The boxes were equipped with 5 nose-poke modules on the back wall and with a magazine at the front wall. During the task, rats had to nose-poke into that hole where the light was turned on. After 5 s inter-trial interval, in one randomly selected nose-poke module a 1 sec long stimulus was presented. The animal made a correct response if nose-poked into this hole during the stimulus presentation or within 5 s afterwards (limited hold). Correct responses were rewarded with a pellet delivered into the magazine. Nose-poke into the magazine initiated the next trial. The animal made an incorrect response if nose-poked into one of the holes where the stimulus was not presented. An omission response was recorded when the rat did not make any nose-poke up to the end of the limited hold. Incorrect responses and omissions were followed by 5 s time-out punishment, when the house light was turned off. After the time-out, the house light was set back and the rat could start the next trial by nose-poking into the magazine. The animal made a premature response, if nose-poked into any of the holes during the inter-trial interval. These responses were also punished with time-out. Length of a daily test session was 20 min. Rats were trained for the task in stages with gradually decreased stimulus duration from 30 to 1 s. Animals could step to the next training stage, if they collected at least 40 (EXP1) or 30 rewards (EXP2) during a training session. One animal which did not even reach the 1st stage (learning to use the nosepoke modul) was excluded from the experiment. The outcome parameters were the days needed to complete the final stage and the learning curve plotted as average learning stage in function of training days.

### Western Blot

After the behavioral tests, the animals were decapitated, their brain were removed and both hippocampi were dissected then frozen and stored at −80°C. Hippocampal tissues were homogenized with TissueLyser (Qiagen, Venlo, Netherlands) in lysis buffer containing 200 mM NaCl, 5 mM EDTA, 10 mM Tris, 10% glycerine, and 1 g/ml leupeptin (pH 7.4), supplemented with a protease inhibitor cocktail (cOmplete ULTRA Tablets, Roche, Basel, Switzerland) and PMSF (Sigma, St. Louis, MO, United States). The homogenized lysates were centrifuged twice at 1,500x g and 4°C for 15 min, then the supernatants were collected and their protein concentration was measured by the bicinchoninic acid assay (Thermo Fisher Scientific, Waltham, MA, United States). Equal amount of protein (20 μg) was mixed with Pierce Lane Marker reducing sample buffer (Thermo Fisher Scientific, Waltham, MA, United States), and loaded and separated in a 4–20% precast Tris-glycine SDS polyacrilamide gel (Bio-Rad, Hercules, CA, United States). Proteins were transferred electrophoretically onto a polyvinylidene difluoride membrane (Bio-Rad, Hercules, CA, United States) at 200 mA overnight. Membranes were blocked with 5% nonfat dry milk (Cell Signaling Technology, Leiden, Netherlands) in Tris buffered saline containing 0.05% Tween-20 (0.05% TBS-T; Sigma, St. Louis, MO, United States) at room temperature for 2 h. Membranes were incubated with primary antibodies against PHF-13 (sc32275, 1:1,000, Santa Cruz Biotechnology, Santa Cruz, CA, United States), Tau (sc32274, 1:1,000, Santa Cruz Biotechnology, Santa Cruz, CA, UnitedStates) and β-Amyloid (sc28365, 1:500, Santa Cruz Biotechnology, Santa Cruz, CA, UnitedStates) overnight at 4°C, followed by 2 h incubation at room temperature with anti-mouse HRP-linked secondary antibody. Phospho-Tau protein expression was normalized to the corresponding total protein. β-Actin was used to control for sample loading and protein transfer and to normalize the content of the β -Amyloid. Signals were detected with a chemiluminescence kit (Bio-Rad, Hercules, CA, UnitedStates) by Chemidoc XRS+ (Bio-Rad, Hercules, CA, UnitedStates). The intensity of the samples was measured by Image Lab software (version 4.1, Bio-Rad, Hercules, CA, UnitedStates). Phospho-specific antibody was removed with Restore™ Western Blot Stripping Buffer (Thermo Fisher Scientific, Waltham, MA, UnitedStates) before the incubation of the corresponding total protein antibody.

### Statistics

Group means ± standard error were calculated and significance was determined by unpaired *t*-test (5CSRTT days to complete, NOR discrimination index, PAL, MWM probe trial, WB), paired *t*-test (NOR discrimination index), Fischer exact test (PAL frequency) or repeated measures ANOVA (MWM escape latencies) using the Statistica 13.5.0.17 software package (TIBCO Software Inc.). The sigmoidal fits to the 5CSRTT learning curves were performed by the Origin 2015 software (OriginLab Corporation). In addition, a multivariate ANOVA was performed on the following variables in both experiments: NOR discrimination index, days needed to reach the final stage in the 5-CSRTT, phospho-tau/tau ratio, β-amyloid level (Statistica 13.5.0.17).

## Results

### Novel Object Recognition

**FIGURE 2:**
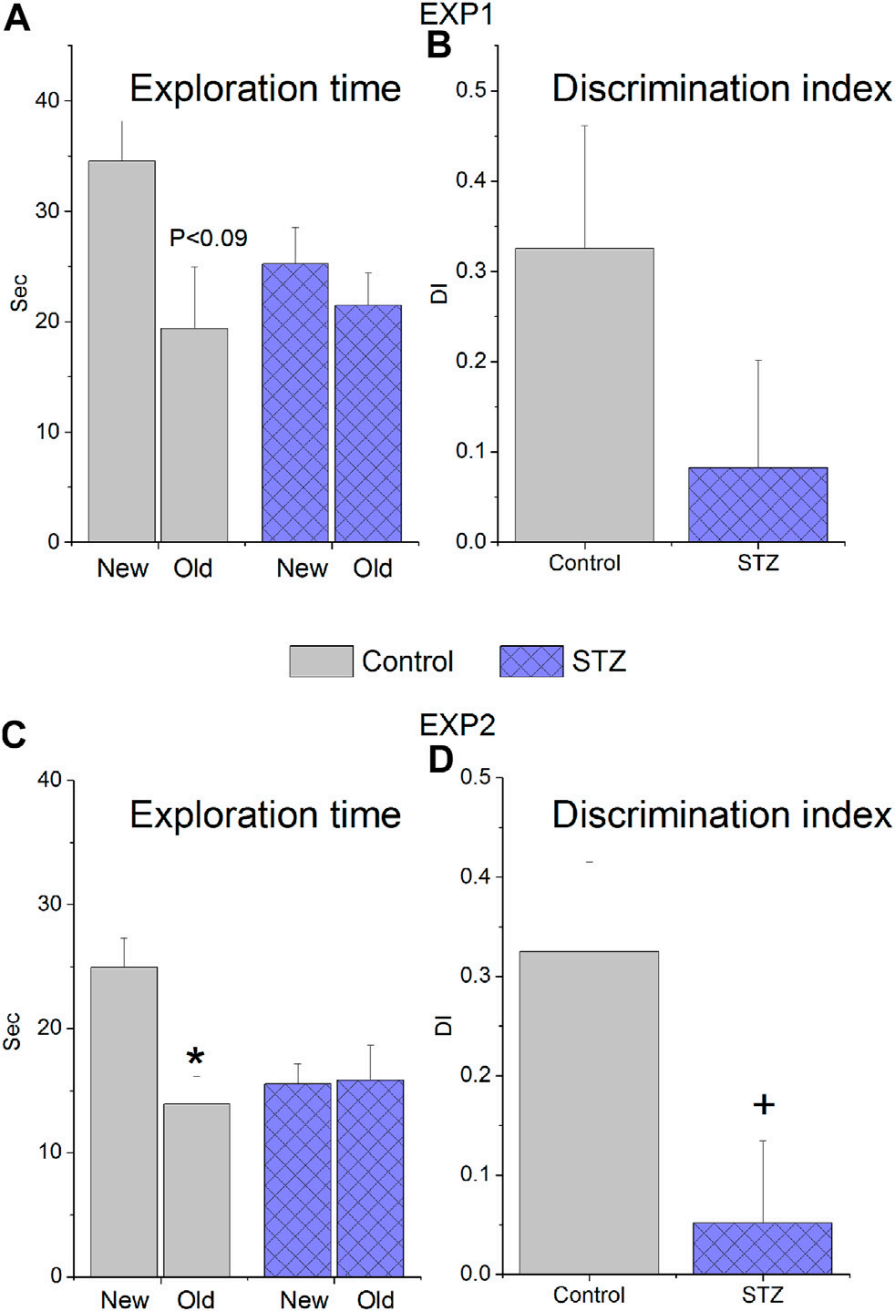
Novel Object Recognition performance of rats treated with icv. streptozotocin (STZ) or citrate buffer (control). Means ± SEM values are shown. **(A,B)** Results of experiment 1 (EXP1), when the animals received 2 × 1.5 mg/kg icv. STZ 8 weeks before the test. Exploration time of the familiar (old) and unfamiliar (new) objects marginally significantly differ in the control group (paired *t*-test: *t*(5) = 2.10, *p* = 0.089) but not in the STZ group (paired *t*-test: *t*(7) = 0.70, ns) and the calculated discrimination indices (DI) did not significantly differ (unpaired *t*-test: *t*(12) = 1.33, ns) **(C,D)** Results of EXP2, when the animals received 3 × 1.5 mg/kg icv. STZ 14 weeks before the test. The control group explored the new object for significantly longer time than the old one (paired *t*-test: *t*(10) = 3.53, *p* < 0.005) while the STZ-treated rats spent equal time in examining the objects (paired *t*-test: *t*(9) = −0.10, ns). The discrimination indices (DI) of the two groups were also significantly different (unpaired *t*-test: *t*(19) = 2.21, *p* < 0,05) *p < 0.05 vs “new”, + < *p* < 0.005 vs “control”.

In EXP1, STZ-treated animals explored less the unfamiliar new object than the control rats (Figure 2A) and their DI value was also much lower (0.33 and 0.08 in the control and STZ group, respectively, Figure 2B), nevertheless, due to the low number of animals remained in the experiment (n = 6 and n = 8 for control and STZ, respectively) the difference was not statistically significant (Figure 2B). In EXP2, control animals spent significantly more time in examining the unfamiliar object (24.9 s) than the old one (13.9 s) whereas STZ-treated rats equally explored both (15.6 s and 15.8 s for new and old, respectively) (Figure 2C). The DI values of the two groups (0.32 and 0.05 for control and STZ, respectively, Figure 2D) were significantly different.

### Passive Avoidance Learning

**FIGURE 3:**
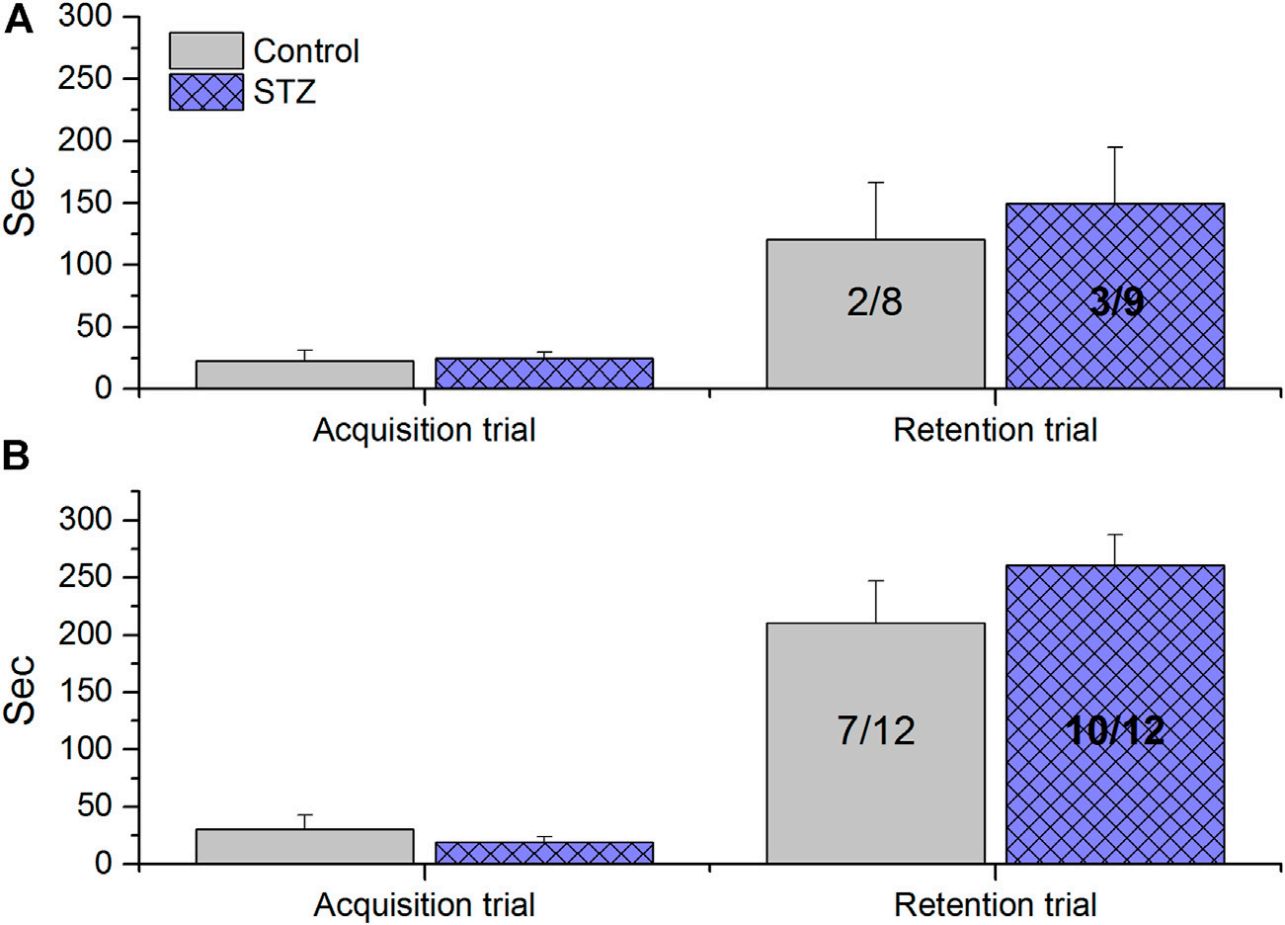
Passive Avoidance Learning results of rats treated with icv. streptozotocin (STZ) or citrate buffer (control). Columns show means ± SEM values of entry latencies, numbers inside the columns indicate the not entered/total number of animals. **(A)** Results of experiment 1 (EXP1), when the animals received 2 × 1.5 mg/kg icv. STZ 11 weeks before the test. No significant difference was observed between control and STZ-treated animals (unpairedt-test acquisition trial: *t*(15) = −0.23, ns; unpaired *t*-test retention trial: *t*(15) = −0.45, ns; Fischer exact p, two tailed test *p* = 1.0, ns). **(B)** Results of experiment 2 (EXP2), when the animals received 3 × 1.5 mg/kg icv. STZ 6 weeks before the test. No significant difference was observed between control and STZ-treated animals (unpaired *t*-test acquisition trial: *t*(22) = 0.85, ns; unpaired *t*-test retention trial: *t*(22) = −1.10, ns; Fischer exact two tailed test *p* = 3707).

There was no significant difference between the learning performances of groups either in acquisition or retention trials in any of the experiments (Figures 3A,B).

### Morris Water-Mate

**FIGURE 4:**
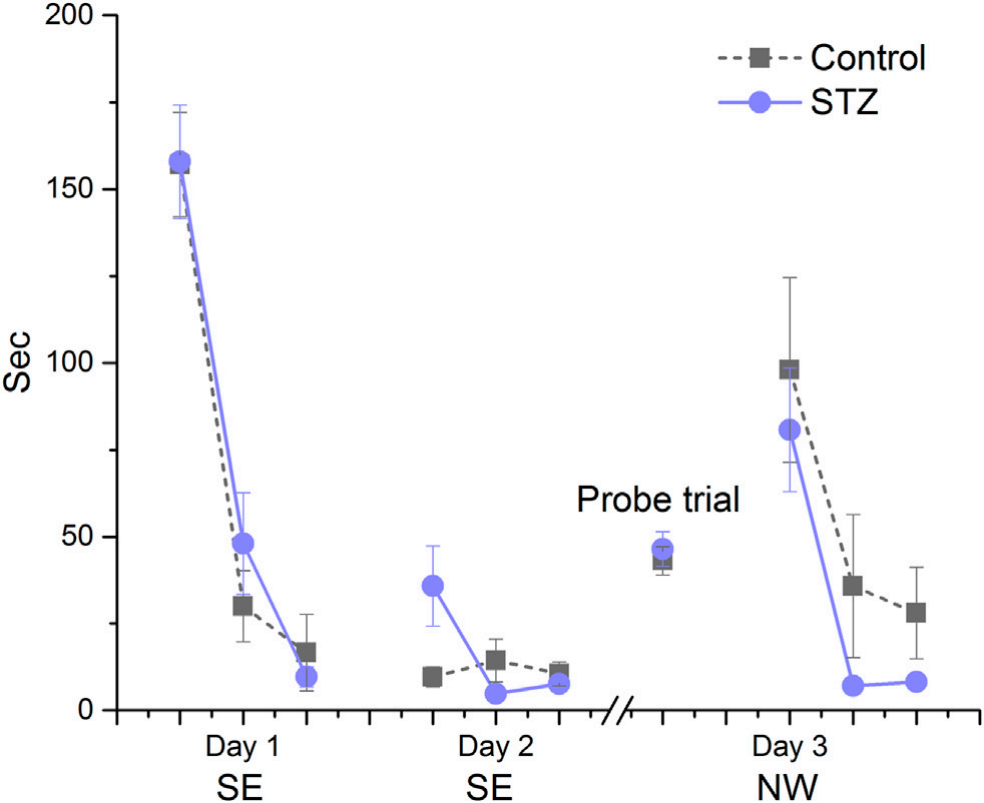
Learning performance in the Morris water-maze in EXP1. Means ± SEM of escape latency values are shown except in the probe trial where the time spent in the target quadrant is depicted. SE and NW indicate the position of the escape platform. There was no significant difference between groups in the acquisition trials on days 1 and 2 (group effect: F_(1,15)_ = 0.89, ns; Day × trial × treatment interaction: F_(2,30)_ = 1.94, ns), in the probe trial (unpaired *t*-test: *t* (15) = −0.51, ns) and during re-acqusition on Day 3 (group effect: F_(1,15)_ = 1.60, ns, Day × treatment interaction: F_(2,30)_ = 0.11, ns).

**FIGURE 5:**
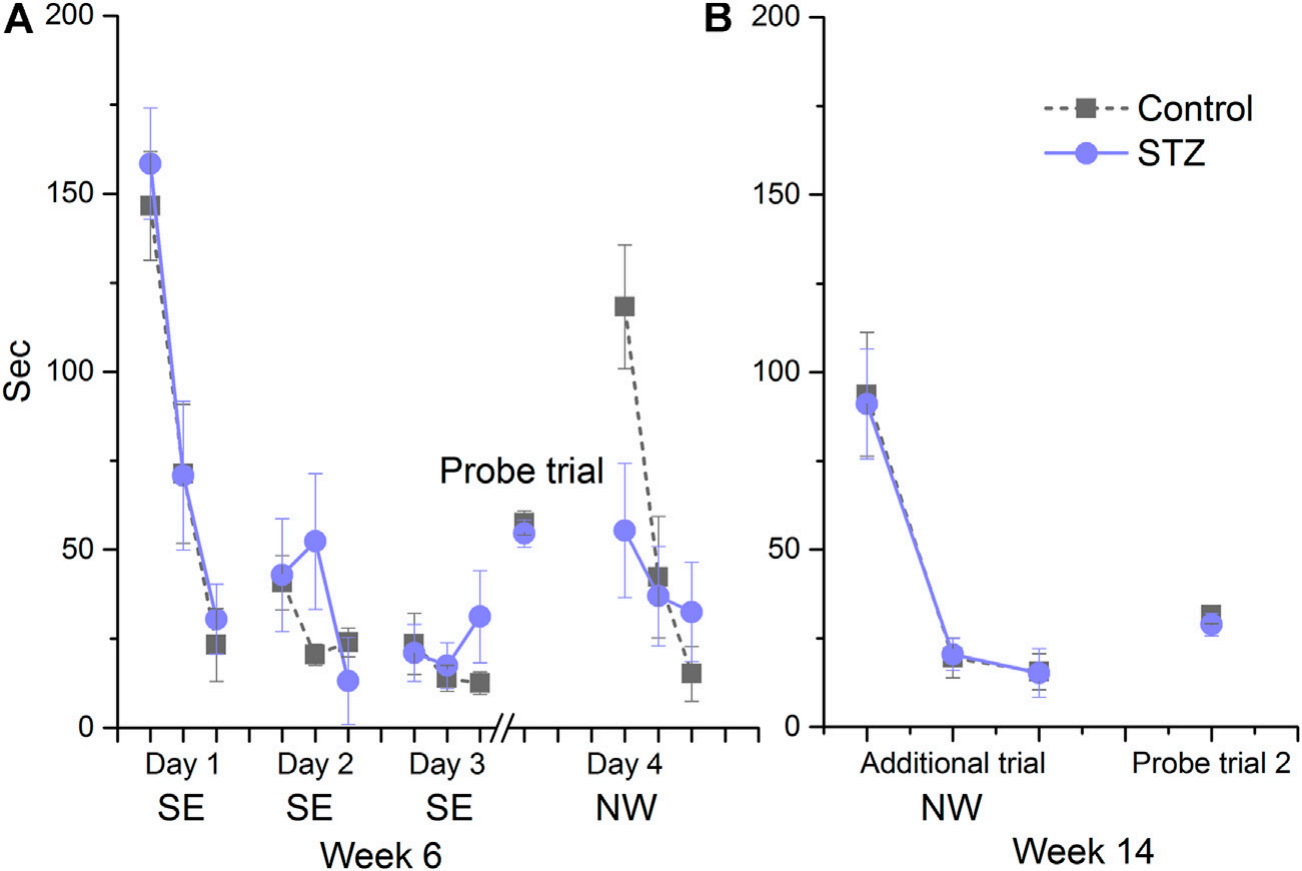
Learning performance in the Morris water-maze in EXP2. Means ± SEM of escape latency values are shown except in the probe trial where the time spent in the target quadrant is depicted. SE and NW indicate the position of the escape platform. **(A)** There was no significant difference between groups in the acquisition trials on days 1, 2 and 3 (Day x trial × treatment interaction: F_(4,88)_ = 1.80, ns) in the probe trial (unpaired *t*-test: *t*(22) = 0.58, ns)) and during the re-acquisition at day 4 (Day × treatment interaction: F_(2,44)_ = 3.79, ns). **(B)** No significant difference was detected during the additional acquisition session (Day × treatment interaction: F_(2,44)_ = 0.01, ns) and the second probe trial (unpaired *t*-test: *t*(22) = 0.66, ns).

In EXP1 this assay was carried out at week 14. Control and treated animals similarly performed in the acquisition trials (days 1–2, Figure 4). All of the rats successfully learned the location of the hidden platform with similar decrease in their escape latency. The animals spent the same amount of time in the target quadrant during the probe trial, furthermore no significant difference was found between the groups during the re-acquisition trials when the platform was replaced to a new location (Figure 4). In EXP2, MWM performance was first measured at week 6 (Figure 5A). Again, no significant difference was detected in the performance of the control and STZ-treated groups in the three phases of the test. To examine the possible later development of cognitive impairment, an additional acquisition session and probe trial were carried out at week 14; nonetheless there was no significant difference between the groups (Figure 5B)*.*

### 5-Choice Serial Reaction Time Task

**FIGURE 6:**
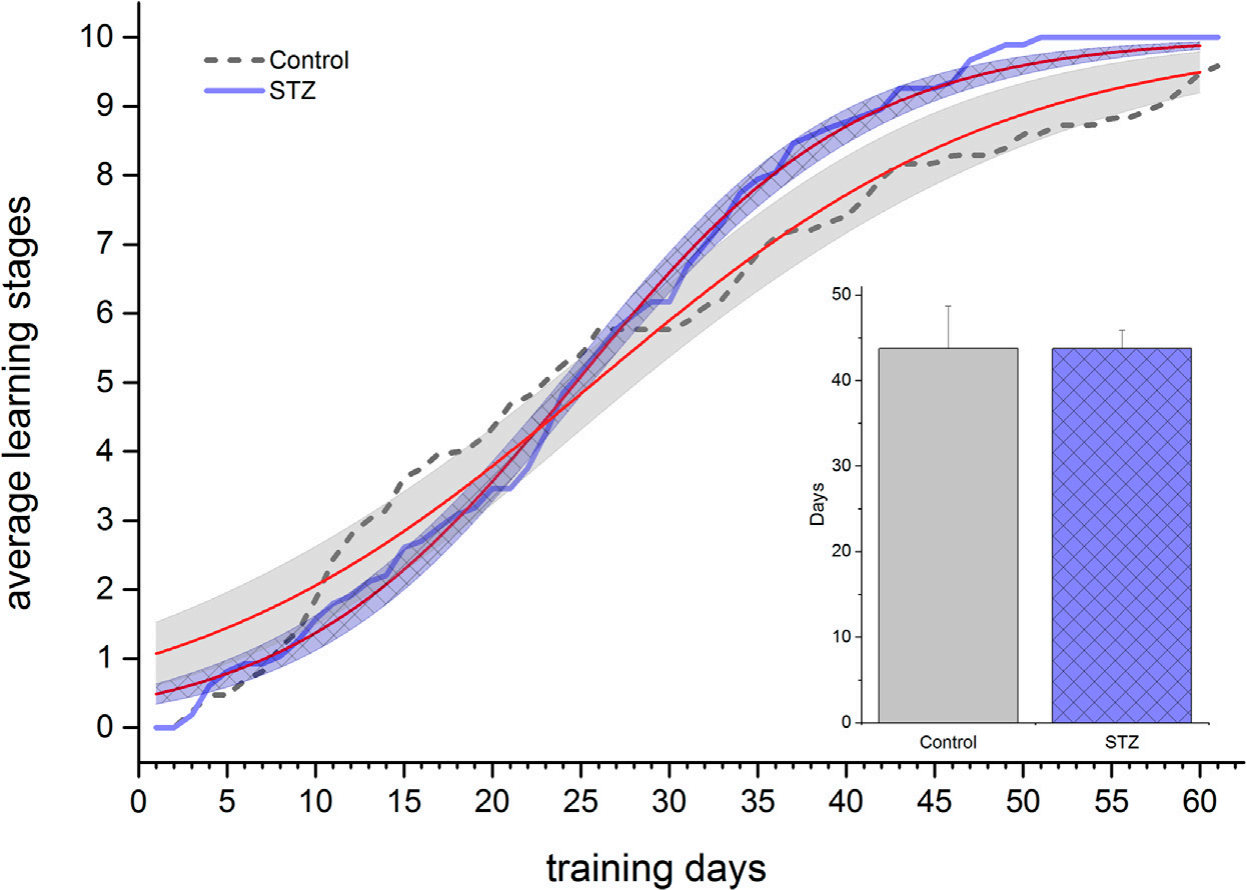
Learning performance in the 5 Choice Reaction Time task in EXP1. Learning curves of control and STZ-treated animals are depicted. Shaded areas show the 95% confidence band of the fitted sigmoidal regression curves (thin [red] lines). The column chart inset shows the number of days taken to reach the maximum stage. Means ± SEM values are shown. No significant difference was detected either between the learning curves or in the days elapsed until reaching the final stage (unpaired *t*-test: *t*(15) = 0.25, ns).

**FIGURE 7:**
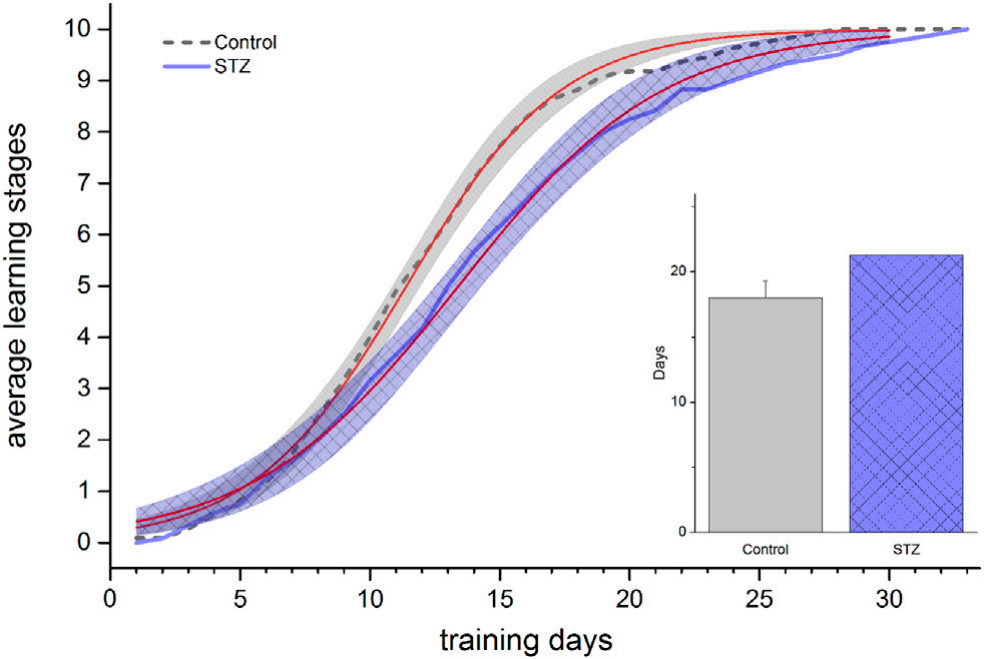
Learning performance in the 5 Choice Serial Reaction Time task in EXP2. Learning curves of control and STZ-treated animals are depicted. Shaded areas show the 95% confidence band of the fitted sigmoidal regression curves (thin [red] lines). The regression lines significantly differ (no overlap between the confidence bands). The column chart inset shows the number of days taken to reach the final stage. Means ± SEM values are shown. No significant difference was detected between the two groups (unpaired *t*-test: *t*(21) = −1.46, ns).

In EXP1, STZ-treated and control animals showed intersecting and overlapping learning curves (inflection points of the fitted sigmoid regression curves were 24.7 and 25.8 days, respectively) and the days needed to reach the maximum learning stage were the same (43.8 and 43.8 days, respectively) (Figure 6). In EXP2, however, the control group learned significantly faster shown by the two days difference in the midpoint of the fitted sigmoid regression curves (11.4 and 13.4 days in the control and STZ-treated group, respectively, Figure 7), furthermore, the STZ-treated animals needed 3 days more to complete the task (21.2 *vs* 18.0 days in the control group), though this difference was not significant.

### Western Blot Measurements

**FIGURE 8:**
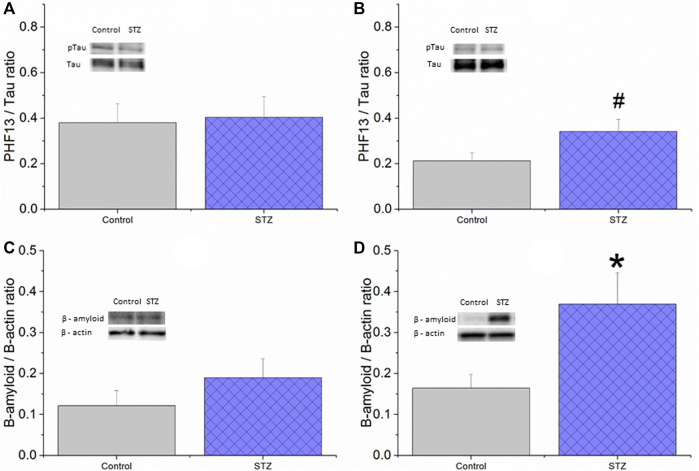
The effect of icv. STZ or citrate buffer (control) treatment on the tissue protein levels of phospho-Tau **(A,B)** and β-amyloid **(C,D)** in EXP1 **(A,C)** and EXP2 **(B,D)** measured by Western blot. Means ± SEM values are shown. There was no significant difference between the groups in phospho-tau/tau ratio in EXP1 (unpaired *t*-test: *t* (14) = −0.19, ns) **(A)** whereas a trend for an increase in the STZ group can be seen in EXP2 (unpaired *t*-test: *t* (22) = −2.012, *p* = 0.06) **(B)** Also, there was no significant difference in β-amyloid level in EXP1 (unpaired *t*-test: *t* (13) = −1.13, ns) **(C)** while significantly elevated β-amyloid level was found in the STZ-treated group in EXP2 (unpaired *t*-test: *t* (20) = −2.45, *p* < 0.05 **(D)** *<0.05, ^#^<0.10.

In EXP1, Western blot analysis revealed no significant difference in phospho-Tau/Tau ratio and β-amyloid level between vehicle- and STZ-treated animals (Figures 8A,C). In EXP2 we found a marginally significant elevated phospho-tau/tau ratio (Figure 8B significant increase in the β -amyloid level in the STZ-treated animals (Figure 8D).

### Multivariate Analysis of Variance

We found more pronounced effects in four out of the six assays in EXP2 vs EXP1, although in themselves they were not always statistically significant. To statistically analyze the overall difference between the two experimental protocols we performed a multivariate ANOVA on four variables each from one of these 4 assays: phospho-tau/tau ratio, β-amyloid level, NOR discrimination index, and days needed to reach the final stage in the 5-CSRTT. The difference between the control and STZ groups was significant in EXP2 (Wilks λ = 0.397, F(4,13) = 4,931; *p* = 0.012) whereas it was not significant in EXP1 (Wilks λ = 0.583, F(4,6) = 1.072; *p* = 0.446).

## Discussion

The icv. STZ-induced brain pathology has been an increasingly used model of Alzheimer’s disease. The preferred subjects of the model are Wistar and to a lesser extent–the Sprague-Dawley rats and mice. A few papers were published on Lewis rats (Blokland and Jolles, 1993; Bloch et al., 2017) but pigmented rats–up to our knowledge have not been examined in the model yet. However, apart from the species and strain, several variations of other parameters of the model have been published which offered various options to choose while transferring the model to the Long-Evans strain.

1. Dosing of STZ: the most common dosing is 3 mg/kg split into two 1.5 mg/kg doses injected with two days difference (Sonkusare et al., 2005; Pathan et al., 2006; Prakash and Kumar, 2009; Salkovic-Petrisic et al., 2011, Salkovic-Petrisic et al., 2015; Hashemi-Firouzi et al., 2018; Knezovic et al., 2018; Majkutewicz et al., 2018; Yamini et al., 2018) but single 1 mg/kg (Salkovic-Petrisic et al., 2006, 2015; Grünblatt et al., 2007), 1.5 mg/kg (Blokland and Jolles, 1993; Jee et al., 2008; Deng et al., 2009), 2 mg/kg (Moreira-Silva et al., 2018) and 3 mg/kg (Rodrigues et al., 2010; Correia et al., 2013; Osmanovic Barilar et al., 2015; Samy et al., 2016; Zappa Villar et al., 2018; Bavarsad et al., 2020) doses are also applied. We chose the first regimen.

2. Follow-up period after the injection: the majority of the published studies applied a one month long post-injection period (Li et al., 2012; Correia et al., 2013; Zhou et al., 2013; Hashemi-Firouzi et al., 2018; Zappa Villar et al., 2018) or even shorter, 2–3 weeks intervals (Blokland and Jolles, 1993; Sonkusare et al., 2005; Jee et al., 2008; Deng et al., 2009; Yamini et al., 2018). There are some studies where longer, 3 months follow-up periods were used (Salkovic-Petrisic et al., 2006, Salkovic-Petrisic et al., 2011, Salkovic-Petrisic et al., 2015; Knezovic et al., 2015; Samy et al., 2016; Knezovic et al., 2018; Ilieva et al., 2019; Voronkov et al., 2019) In two very much informative longitudinal studies (Knezovic et al., 2015; Osmanovic Barilar et al., 2015) changes/impairments were followed up to 9 months. In our study we chose a 3–3.5 months follow up period considering that the method is intended to be a model of Alzheimer’s disease, which would imply slowly evolving long term pathological changes and that it allowed to conduct learning tasks requiring several weeks training, like 5-CSRTT.

3. Selected cognitive assays and their timing: PAL (fear memory) and MWM (spatial learning and memory) are by far the most common tasks in the literature with usually significant impairments in the STZ-treated groups. Their timing varies but impairments were shown already at 2–3 weeks post-injection both in the PAL (Blokland and Jolles, 1993; Lannert and Hoyer, 1998; Sharma and Gupta, 2001; Sonkusare et al., 2005; Pathan et al., 2006; Jee et al., 2008; Knezovic et al., 2015; Samy et al., 2016) and in the MWM assays (Pathan et al., 2006; Grünblatt et al., 2007; Prakash and Kumar, 2009; Agrawal et al., 2011; Zhou et al., 2013; Salkovic-Petrisic et al., 2015; Samy et al., 2016; Rajasekar et al., 2017; Majkutewicz et al., 2018; Yamini et al., 2018). Later measurements (1–3 months) also showed impaired performance; PAL: (Samy et al., 2016; Lu et al., 2017; Hashemi-Firouzi et al., 2018; Knezovic et al., 2018; Bavarsad et al., 2020), MWM: (Shoham et al., 2003; Salkovic-Petrisic et al., 2006; Grünblatt et al., 2007; Rodrigues et al., 2010; Li et al., 2012; Correia et al., 2013; Salkovic-Petrisic et al., 2015; Samy et al., 2016; Knezovic et al., 2018; Bavarsad et al., 2020; Liu et al., 2020). Impaired visual recognition memory was also detected in the novel object recognition paradigm 3–8 weeks after STZ-treatment. We chose to apply these cognitive assays. To extend the cognitive domains under investigation we added the 5-CSRTT paradigm (attention). This task requires a long training period therefore we started with it soon after recovery from surgery in EXP1. Timing of the other three assays was based on literature data with taking care to avoid interference with the initial training phase of the 5-CSRTT.

4. Detecting amyloid and tau pathology: increase in phospho-tau/tau ratio was already observed from 2 weeks post-injection and was detected either by Western-blot technique (Salkovic-Petrisic et al., 2006; Grünblatt et al., 2007; Li et al., 2012; Correia et al., 2013; Kosaraju et al., 2013; Zhou et al., 2013; Osmanovic Barilar et al., 2015; Salkovic-Petrisic et al., 2015; Lu et al., 2017; Moreira-Silva et al., 2018; Zappa Villar et al., 2018) or by immunostaining (Lu et al., 2017; Knezovic et al., 2018; Wu et al., 2018). Increase in β-amyloid was shown at later timepoints, about 1.5 months on, by either ELISA (Correia et al., 2013; Kosaraju et al., 2013; Samy et al., 2016; Lu et al., 2017; Wu et al., 2018; Ilieva et al., 2019) or Western-blot (Choi et al., 2014; Kang and Cho, 2014; Zappa Villar et al., 2018) or immunostaining (Salkovic-Petrisic et al., 2006; Choi et al., 2014; Kang and Cho, 2014; Knezovic et al., 2015; Salkovic-Petrisic et al., 2015). Amyloid-plaque like deposits appeared first at 3 months after STZ injection in the meningeal vessels visualized either by congo red (Salkovic-Petrisic et al., 2006) or by immunostaining (Bloch et al., 2017). At 6 and 9 months they became more pronounced (Salkovic-Petrisic et al., 2011) and progressed into the brain parenchyima (Knezovic et al., 2015). We chose Western blot detection of both phospho-tau and β-amyloid proteins.

During EXP1, we couldn’t find any significant difference between the control and STZ-treated groups either in the behavioural assays or in the histological markers β-amyloid and phospho-tau/tau ratio. STZ animals learnt the MWM and 5-CSRTT tasks as well as control animals did. In the PAL test relatively low memory trace could be observed even in the control group. In the NOR assay the control animals showed a sufficient level of discrimination while the STZ-treated rats were much inferior, but due to the low final sample size these differences were not significant.

The results obtained during EXP1 suggested that the dose of STZ may have been inadequate in Long-Evans rats. Therefore, we increased the dose by a factor of 1.5 in EXP2. It was a cautious increase since the exact dose-response relationship is not entirely clear for the icv STZ, and some reports showed dramatic changes even at the 3 mg/kg dose (Bloch et al., 2017). Furthermore, personal communications on unpublished experimental attempts also warned us about severe histological or behavioural toxicity. Instead of increasing the injected dose we added a third 1.5 mg/kg injection partly to avoid acute toxicity, partly to approximate a more prolonged STZ influence.

We also changed the timing of the cognitive assays. We assumed that the early 5 CSRTT learning engagement and the consequential frequent handling of the animals may have had a protective effect against STZ treatment. During EXP2 we dismissed any measurements in the first and a half month to allow a kind of incubation period. PAL and MWM, as the most sensitive tests were the first, while the 5-CSRTT training started afterwards to avoid the above mentioned interference. The NOR test was placed to the end. However, as we did not get any impairment in the MWM, we repeated it at the end of the follow-up period to see if the deterioration could be detectable by then.

During EXP2, we could not again find significant difference in the PAL and MWM tests. In the former, the observed memory trace in the control group was good enough this time to allow to detect an eventual inhibition, yet STZ treated animals performed at least as well as the controls. In the MWM tests, animals showed a similar performance both in the acquisition and probe trials both at the first and the second occasion. These results are in sharp contrast to the findings of the literature referred above, and the discrepancy is not easily explainable. For the MWM one may speculate that STZ treatment may affect visual acuity, which plays an important role in MWM learning, and the superior visual acuity of pigmented rats over white ones (Prusky et al., 2002) may have remained more functional after the STZ-treatment. In most of the cited studies, white STZ-treated rats also showed a learning process but slower than the controls. In the studies of (Prakash and Kumar, 2009; Prakash et al., 2015) STZ-treated animals also found a visible platform significantly slower than the controls. These findings suggest that the impaired MWM performance may resulted from–at least partly–a visual impairment. Certainly, such a difference cannot play a role in the PAL task. In this assay a possible–though admittedly feeble–explanation could be if STZ would cause a higher anxiety state in Long-Evans than in white rats, which would resulted in a higher sensitivity to punishment allowing stronger fear memory formation.

In the NOR test, control but not STZ-treated animals explored significantly more the unfamiliar object, and the discrimination index of the STZ group was significantly lower. These findings are in accordance with those in the literature (Lu et al., 2017; Hashemi-Firouzi et al., 2018; Moreira-Silva et al., 2018; Wu et al., 2018; Zappa Villar et al., 2018). In the 5-CSRTT paradigm STZ-treated animals showed slower learning than the controls, although they were also able to acquire the task. In the Western blot measurements we found marginally significant increase in the phospho-tau/tau ratio and significant increase in the β-amyloid level in the hippocampus of animals in the STZ group compared to controls. These results are again in line with those of the literature (Correia et al., 2013; Kosaraju et al., 2013; Samy et al., 2016; Lu et al., 2017; Wu et al., 2018; Zappa Villar et al., 2018) and point out that the 3 × 1.5 mg/kg dose was sufficient to induce biochemical changes.

Overall, in EXP2, the effects of STZ were more pronounced in the NOR, 5-CSRTT, β-amyloid, and phospho-tau assays compared to EXP1, which was confirmed by the multivariate analysis. However, we still get no difference in the two key tests, MWM and PAL. We can conclude that some tests may be more sensitive to treatment (prominently the NOR task), while the aversively motivated learning tasks (PAL and MWM) still remained insensitive to the effect of STZ despite the elevated dose. Thus, our findings suggest that Long-Evans rats are likely less sensitive to STZ treatment. As this strain is crucial in our test system, we continue experimenting in it with the STZ treatment. We plan to apply the 3 × 1.5 mg/kg dosing of STZ in trained, experienced animals and also in aged rats. A possible modification of the model could be the injection of 3 × 2 mg/kg dose or administration of STZ via osmotic minipump, to ensure a continuous and longer lasting exposure to the drug.

## Data Availability

The raw data supporting the conclusions of this article will be made available by the authors, without undue reservation.
