# Corrigendum: Intracerebroventricularly Injected Streptozotocin Exerts Subtle Effects on the Cognitive Performance of Long-Evans Rats

**DOI:** 10.3389/fphar.2022.1122260

**Published:** 2023-01-05

**Authors:** Attila Gáspár, Barbara Hutka, Aliz Judit Ernyey, Brigitta Tekla Tajti, Bence Tamás Varga, Zoltán Sándor Zádori, István Gyertyán

**Affiliations:** Department of Pharmacology and Pharmacotherapy, Semmelweis University, Budapest, Hungary

**Keywords:** Alzheimer disease model, STZ icv., cognitive test battery, learning impairment, β-amyloid, phospho-tau

In the published article, there was an error in Figure 8 as published. The results of a mistaken measurement were shown in Figures 8A, B. Consequently, the numerical values of the *t*-test comparing the phospho-tau/total tau ratios in the control and STZ treated groups in EXP1 and EXP2 are inadequate**.** The corrected Figure 8 and its corrected caption appear below:

**FIGURE 8:**
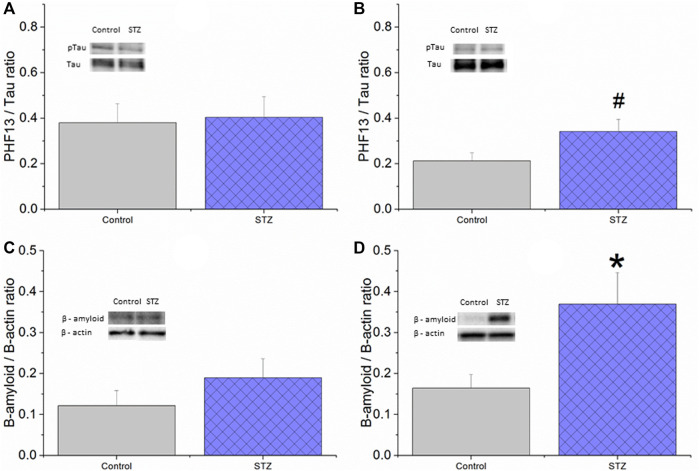
The effect of icv. STZ or citrate buffer (control) treatment on the tissue protein levels of phospho-Tau **(A,B)** and β-amyloid **(C,D)** in EXP1 **(A,C)** and EXP2 **(B,D)** measured by Western blot. Means ± SEM values are shown. There was no significant difference between the groups in phospho-tau/tau ratio in EXP1 (unpaired *t*-test: t (14) = −0.19, ns) **(A)** whereas a trend for an increase in the STZ group can be seen in EXP2 (unpaired *t*-test: t (22) = −2.012, *p* = 0.06) **(B)** Also, there was no significant difference in β-amyloid level in EXP1 (unpaired *t*-test: t (13) = −1.13, ns) **(C)** while significantly elevated β-amyloid level was found in the STZ-treated group in EXP2 (unpaired *t*-test: t (20) = −2.45, *p* < 0.05 **(D)** *<0.05, #<0.10.

Furthermore, the name and catalogue number of the phospho-tau antibody in the Western Blot section was erroneous. As such, a correction has been made to “Methods and materials, Western Blot.” The sentence previously stated:

“Membranes were incubated with primary antibodies against PHF1 (sc515013, 1:1,000, Santa Cruz Biotechnology, Santa Cruz, CA, United States)…”

The corrected sentence appears below:

“Membranes were incubated with primary antibodies against PHF-13 (sc32275, 1:1,000, Santa Cruz Biotechnology, Santa Cruz, CA, United States)…”

The numerical values of the multivariate analysis of variance results were also inadequate. Therefore, a correction has been made to “Results, Multivariate analysis of variance.” The sentence previously stated:

“The difference between the control and STZ groups was significant in EXP2 (Wilks λ = 0.391, F(4,13) = 5.054; *p* = 0.011) whereas it was not significant in EXP1 (Wilks λ = 0.750, F(4,7) = 0.583; *p* = 0.685298344).”

The corrected sentence appears below:

“The difference between the control and STZ groups was significant in EXP2 (Wilks λ = 0.397, F(4,13) = 4,931; *p* = 0.012) whereas it was not significant in EXP1 (Wilks λ = 0.583, F(4,6) = 1.072; *p* = 0.446).”

The authors apologize for these errors and state that this does not change the scientific conclusions of the article in any way. The original article has been updated. This is a provisional file, not the final typeset article

